# Plasminogen-Loaded Fibrin Scaffold as Drug Delivery System for Wound Healing Applications

**DOI:** 10.3390/pharmaceutics14020251

**Published:** 2022-01-21

**Authors:** Tamer Al Kayal, Marianna Buscemi, Aida Cavallo, Ilenia Foffa, Giorgio Soldani, Paola Losi

**Affiliations:** 1 Institute of Clinical Physiology, National Research Council (CNR), 54100 Massa, Italy; atamer@ifc.cnr.it (T.A.K.); marianna.buscemi@ifc.cnr.it (M.B.); aida.cavallo@ifc.cnr.it (A.C.); ilenia@ifc.cnr.it (I.F.); giorgio.soldani@ifc.cnr.it (G.S.); 2 Institute of Life Sciences, Scuola Superiore Sant’Anna, 56127 Pisa, Italy

**Keywords:** fibrin scaffold, plasminogen, scratch test, diabetic mice, histological analysis, wound healing

## Abstract

Plasminogen is a protein involved in intravascular and extravascular fibrinolysis, as well as in wound healing, cell migration, tissue formation and angiogenesis. In recent years its role in healing of tympanic perforations has been demonstrated in plasminogen deficient mice. The aim of this work was to fabricate a fibrin-based drug delivery system able to provide a local and sustained release of plasminogen at the wound site. Initially, the biological activity of plasminogen was evaluated by in vitro experiments on cell cultures. A metabolic assay (MTT) was carried out on L929 mouse fibroblast to determine the concentration that does not affect cell viability, which turned out to be 64 nM. The effect of plasminogen on cell migration was evaluated through a scratch test on human keratinocytes: cells treated with 64 nM plasminogen showed faster scratch closure than in complete medium. Fibrin scaffold loaded with plasminogen was fabricated by a spray process. SEM analysis showed the typical nano-fibrillar structure of a fibrin scaffold. Tensile tests highlighted significantly higher value of the ultimate stress and strain of fibrin scaffold with respect to fibrin clot. The in-vitro release kinetic showed an initial plasminogen burst, after that the release slowed, reaching a plateau at 7 days. Plasminogen-loaded fibrin scaffold applied in full-thickness diabetic mouse lesions showed a significantly higher closure rate at 14 days than scaffold used as a reference material. Histological analysis demonstrated an improved reepithelization and collagen deposition in granulation tissue in mouse treated with plasminogen-loaded fibrin scaffold in comparison to unloaded fibrin scaffold. The obtained results demonstrated the suitability of the fibrin scaffold loaded with plasminogen as drug delivery system and suggest its use in wound healing applications, such as for the treatment of chronic diabeticulcers.

## 1. Introduction

Plasminogen (Plg) is a glycoprotein involved in intravascular and extravascular fibrinolysis (extracellular proteolytic system), as well as in wound healing, cell migration, tissue formation and angiogenesis [1]. An impairment in fibrin cleaning and wound healing, due to low level of Plg in the body, results in a cycle with triggered inflammation, leading to the membranes becoming increasingly thick. Plg is converted into plasmin by the cleavage of serine proteases: tissue-type plasminogen activator (t-PA) or urokinase-type plasminogen activator (u-PA). Plg is involved in fibrinolysis, when it is activated by t-PA. However, if it is activated by u-PA, Plg is involved in wound healing and tissue formation. The actual substrate of plasmin, which is fibrin, serves to inhibit excessive clotting [1].

It has been highlighted the importance of Plg activation system not only in hemostasis but also in other processes such as wound healing, in 2011 Juncker-Jensen and Lund demonstrated that wound healing in Plg-deficient mice is significantly delayed respect to wild-type mice [2]. The epidermal covering in wild-type mice was 15.6 days, while Plg-deficient mice had a mean healing time that was delayed to 34.1 days.

The key role of Plg has been demonstrated in tympanic membrane perforation. The healing process in wild type control mice was completed within 8–11 days after the perforation, while the healing was totally arrested in Plg-deficient mice [3]. The healing of tympanic membrane perforations is significantly delayed in mice deficient in u-PA but normal in t-PA deficient mice. Keratinocyte migration, fibrin clearance and the resolution of inflammation are delayed in u-PA mice [4]. More recently it has been demonstrated that daily local injection of Plg into the soft tissue surrounding the tympanic membrane restored the ability to heal tympanic perforations in Plg deficient mice in a dose-dependent manner and potentiates the healing in wild-type mice [5].

Fibrin represents a natural biopolymer naturally involved in wound healing. In fact, beyond its role in hemostasis, it acts as a local reservoir for growth factors and as a provisional matrix for several cell types that drive the regenerative process. Furthermore, fibrin contains Arg-Gly-Asp (RGD) sites that serve as recognition motifs for endothelial cells and fibroblasts. ECM proteins, such as fibronectin, vitronectin or thrombospondin, bind fibrin and effectively regulate cell adhesion, proliferation and migration; for example, the influx of monocytes or fibroblasts during the granulation process [6]. For this characteristic, fibrin, in a different form such as fibrin glue [7], platelet rich fibrin [8], fibrin scaffold seeded with keratinocytes and fibroblasts or growth factors [9], was used in wound healing.

Angiogenesis is key to wound healing and fibrin-based delivery systems have an inherent advantage in that fibrin intrinsically stimulate angiogenic activity. VEGF and FGF have been incorporated into fibrin matrices and successfully delivered to enhance angiogenesis [10,11]. Fibrin matrices are susceptible to proteolysis in vivo, especially by plasmin that solubilize matrix proteins and release the entrapped molecules.

In this work a fibrin-based drug delivery system has been described to obtain a local and sustained release of Plg at the wound site. Fibrin scaffold loaded with Plg was fabricated by a peculiar spray technique that allows for obtaining a thin, resistant handling scaffold. Morphological, mechanical and biological characterization were carried out to evaluate the suitability of this scaffold for wound healing applications, such as for the treatment of chronic diabetic ulcers.

## 2. Materials and Methods

### 2.1. Materials

Human lyophilized fibrinogen, thrombin and Plasminogen (Plg) were supplied by Kedrion Biopharma S.p.A. (Castelvecchio Pascoli, Lucca, Italy). Fibrinogen was dissolved in aqueous solution containing 1% l-arginine, 1% l-lysine, and 0.94 PEU/mL aprotinin to obtain a 60 mg/mL solution. Initially, thrombin was reconstituted in 275 mM CaCl_2_ solution at 1250 U/mL, then it was diluted in distilled water to obtain a 625 U/mL solution and lyophilized Plg (2.5 mg/mL) was added to the solution.

### 2.2. Cell Culture Conditions

Mouse fibroblasts cell line L929 (ICLC ATL95001) was obtained from Biobanking and Cell Factory Hospital San Martino (Genova, Italy). Human keratinocytes HaCaT (BS CL 168) were obtained from Biobanking of Veterinary Resources, Istituto Zooprofilattico Sperimentale della Lombardia e dell’Emilia Romagna (Brescia, Italy).

Cells were cultured in RPMI 1640 supplemented with 10% Foetal Bovine Serum (FBS), 2 mM l-Glutamine, 100 μg/mL streptomycin and 100 U/mL penicillin at 37 °C in a uhumidified atmosphere with 5% CO_2_. Medium was routinely changed every 3 days and cells were sub-cultured (1:3 split ratio) at confluence by trypsinization with 0.5% trypsin and 0.02% EDTA. All reagents were purchased from Merck KGaA (Darmstadt, Germany).

### 2.3. Evaluation of Plasminogen Concentration on Cell Viability

Preliminary tests were carried out to identify the non-toxic concentration of Plg by means of MTT (3-(4,5 dimethylthiazol-2-yl)-2,5-diphenyl tetrazolium bromide) test.

The quick and easy method of tetrazolium based colorimetric MTT assay was used to assess cell viability. Briefly, L929 mouse fibroblasts (4 × 10^3^ cells/well) were seeded in triplicate in 96-well plates. After 24 h of incubation, when cell growth was in the log phase, the medium was replaced with 200 μL/well of complete culture medium containing Plg at a concentration of 4, 16 and 64 nM. The complete culture medium and the serum-free medium were used as positive and negative reference controls, respectively. Cells were allowed to grow for another 72 h at 37 °C; after this period 20 μL of an MTT phosphate buffered solution (0.5 mg/mL final concentration) was added to each well and cultures were incubated at 37 °C for 4 h. The supernatant was removed from the wells by slow aspiration and replaced with DMSO (100 μL per plate) to solubilize the MTT tetrazolium dye. At the end of the incubation time, the optical density (OD) was measured at 550 nm wavelength using a microplate reader (Spectrafluor Plus; TECAN Austria GmbH, Grödig, Austria). The percentage of cell viability was calculated vs. the complete culture medium (assumed as 100%).

### 2.4. Evaluation of Released Plasminogen on Cell Migration

Plg effect on wound healing-associated migration was assessed employing the scratch-wounded keratinocyte monolayer models (scratch closure assay). HaCaT were seeded into 24-well plates at a density of 20 × 10^4^, cultured to confluence and scratched by scraping with a 10 μL pipette tip (cell free zone width ~0.8 mm). Culture medium was removed, and cells were fed with 500 μL/well of serum-free medium containing released Plg diluted at different concentration (64 and 640 nM). Control wells received complete culture medium or serum free medium. At 0 and 20 h after scratching, digital images of cells were captured by a phase contrast microscope (Axiovert 25; Zeiss, Milan, Italy; O.M. 50×) equipped with a digital camera (EOS 1000D; Canon, Milan, Italy). Scratch area was measured by an open source image processing program (ImageJ 1.53e software; National Institutes of Health, Bethesda, MD, USA). Migration rate was expressed as percentage of scratch closure compared to the initial area and was calculated as follow: ((At0 − At20)/At0) × 100.

### 2.5. Fabrication of Plasminogen-Loaded Fibrin Scaffold

The Plg-loaded fibrin scaffold was fabricated by a peculiar apparatus named spray-machine previously described [12]. An 8 mL fibrinogen solution (60 mg/mL) was loaded into a 10 mL syringe and 4 mL of Plg-thrombin solution (2.5 mg/mL, 625 U/mL, respectively) in a 5 mL syringe; the solutions were simultaneously sprayed on a rotating metallic mandrel (diameter = 3 cm) at low flow-rates: flow rate of fibrinogen solution was 0.33 mL/min, while Plg-thrombin flow rate was 0.167 mL/min (IT Patent application pending N. 102021000025664).

Then, the Plg-loaded fibrin scaffold was incubated for 1 h in a humidified CO_2_ incubator at 37 °C to achieve a complete fibrin polymerization. After incubation, the scaffold was longitudinally cut and punched to obtain round samples (diameter = 1.5 cm; area = 1.77 cm^2^, containing 0.35 mg of Plg) and placed into a 24-well culture plate. The samples were stored at −80 °C until in vitro or in vivo experiments were performed. Sterile conditions were maintained throughout all manufacturing steps. Plg-unloaded fibrin scaffold was fabricated in the same way as control scaffold without Plg.

### 2.6. Scaffold Morphological Characterization

Plg-loaded fibrin scaffold samples were fixed in 2.5% glutaraldehyde in 0.05 M sodium cacodylate buffer (pH 7.4) for 30 min. Then samples were serially dehydrated for 10 min each in 50%, 70%, 80%, 90% and 100% ethanol in distilled water, for 20 min each in 33%, 67%, 100% hexamethyldisilazane (HMDS; Merck KGaA, Darmstadt, Germany) in ethanol and in 100% HMDS solution until complete evaporation, before sputter-coating with 20 nm gold film. SEM images were taken at an accelerating voltage of 7 kV and 6-mm working distance by an electron microscope (FlexSEM 1000, Hitachi, Tokyo, Japan). Fiber thickness was measured by ImageJ in randomly selected areas for a total of 30 fibers per samples.

### 2.7. Scaffold Mechanical Characterization

Uniaxial tensile test was performed on Plg-loaded fibrin scaffold and on Plg-unloaded fibrin scaffold samples with height of 30 mm and width of 5 mm to investigate the scaffold stiffness. In addition, the test was performed also on samples of fibrin clot obtained by a commercial duploject and a mould with the same size of scaffold samples. Tests were carried on by a mechanical testing system (Z1.0 Zwick GmbH & Co., Ulm, Germany) equipped with a 100 N load cell. Each test was repeated three times with an initial distance between the grippers of 10 mm and a traction speed of 1 mm/min. For each sample, the tensile force and elongation were collected using the TestXpert II V3.41 software (Zwick GmbH & Co., Ulm, Germany) to obtain the stress-strain graph. The Young’s Modulus, for each sample, was calculated considering the slope of the linear part of the stress-strain curve and subsequently averaged over the number of samples. The maximum stress and deformation were also calculated.

### 2.8. Plasminogen Release Kinetic from Plg-Loaded Fibrin Scaffold

The release of Plg from Plg-loaded fibrin scaffold was carried out in PBS at pH 7.4. Scaffold specimens (1 cm^2^) were incubated in 500 µL of PBS under continuous agitation (40 rpm) at 37 °C for 7 days. Daily for four days and at the 7th day, the supernatant was collected and replenished with 500 µL of PBS. The supernatants were stored at −80 °C. Plg quantification was carried on by an ELISA Kit (antibodies-online GmbH, Aachen, Germany) according to manufacturer instructions.

Plg amount (µg) was determined in triplicate. The cumulative release of Plg was calculated by adding the release amount at each time point and expressed as percentage of the total amount of each growth factor loaded in the analyzed scaffold.

### 2.9. Wound Healing Experiments

In total, 20 male diabetic mice (BKS.Cg-m+/+Lepr, db/db) were supplied by Envigo S.r.l. (Udine, Italy) at 10 weeks. Animal care and experimental protocol were approved by the Italian Ministry of Health via decree n. 469/2020-PR, released on 18 May 2020 according to D. lgs. 26/2014. Before administering the experimental protocol, all mice were housed at the Experimental Biomedicine Center of CNR Research Area in Pisa in individual cages on ventilated racks to undergo an acclimatization phase lasting one week to reduce the stress. At each time-point, animals were weighed, and blood glucose levels were measured by a glucometer.

The following described surgical procedure was employed. A single round full-thickness skin wound was created on the back of each animal by a disposable 8 mm diameter skin biopsy punch (Acuderm, Inc, Fort Lauderdale, FL, USA). Animals were randomly assigned to the following groups: 10 mice with Plg-loaded fibrin scaffold (diameter 1.5 cm, thickness ~3 mm, 0.35 mg of Plg/sample); 10 mice with Plg-unloaded fibrin scaffold (negative control). Scaffolds were applied with the nanostructured layer in contact with wound.

All experimental groups were treated with a secondary dressing (transparent dressing polyurethane film Mepore^®^, Mölnlycke Health Care, Gothenburg, Sweden) that serves to keep scaffolds in place and to maintain the wound sterility.

According to the experimental design, at 7 days, under anaesthesia the scaffold was removed, the wounds were photographed, and fresh scaffold was applied to the wounds. At 14 days, the scaffold was removed, and animals were sacrificed by isoflurane inhalation overdose and wounds were photographed before tissue excision. Tissues from the wounded area were fixed in 10% neutral buffered formalin for histological analysis.

### 2.10. Calculation of Wound Area

The healing area was measured according to the previously described protocol [13]. At 0, 7 and 14 days, wounds were photographed, and the wound area was measured by AxioVision Rel. 4.6 software (Carl Zeiss, Oberkochen, Germany). Wound areas were expressed as a percentage of area at day 0: wound area at day X (%) = (wound area at day X/wound area at day 0) per 100.

### 2.11. Histological Analysis of Wound Healing

Samples were embedded in paraffin after dehydration in alcohol series and Xylene and sectioned at 10-µm thick perpendicular to the wound in the central area of the sample. The sections were stained using hematoxylin–eosin (H&E) and Masson’s trichrome with Light Green to evaluate re-epithelialization and collagen deposition. Images were taken with a digital camera (AxioCam 105 Color, Carl Zeiss, Oberkochen, Germany) attached to a light microscope (Axio Zoom.V16, Carl Zeiss, Oberkochen, Germany). For semiquantitative histopathological scoring every parameter was given a score of 0 to 3 based on its level of abundance. Score 0 indicates complete absence, 1 discrete, 2 moderate and 3 profound manifestation of the assessed parameter [14].

### 2.12. Statistical Analysis

All the experiments were carried out at least in triplicate. Data have been presented as mean ± SD. The data were analyzed by StatView 5.0 software (SAS Institute, Cary, NC, USA). The values were statistically compared by parametric (independent Student’s *t*-test) and nonparametric test (Mann–Whitney U test). Values of *p* < 0.05 were considered statistically significant.

## 3. Results

### 3.1. Evaluation of Effect of Plasminogen Concentration on Cell Viability

**Figure 1:**
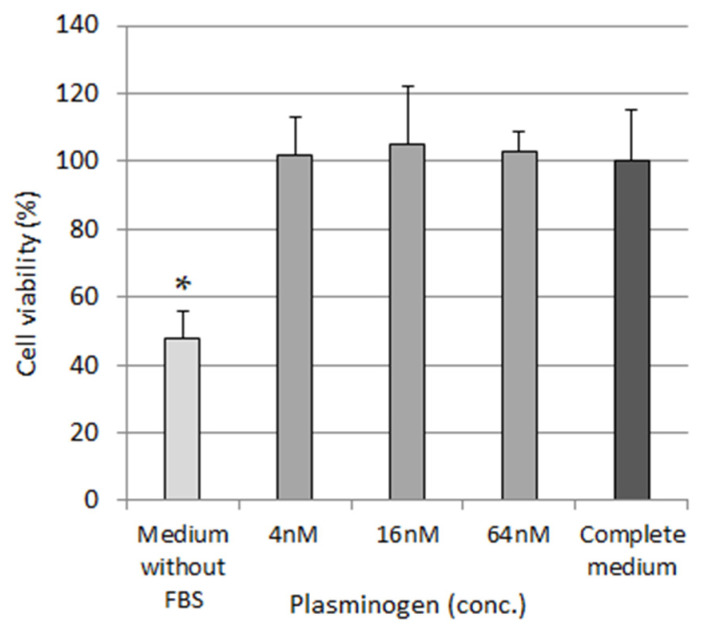
Cytotoxic effects of various Plg concentrations on L929 cells. * *p* ˂ 0.05 vs all samples.

The effect on cell viability of 4, 16 and 64 nM Plg was analyzed against L929 fibroblast cells. The obtained results showed that the cell incubated with all the Plg concentrations induced a cell viability comparable to complete culture medium assumed as 100% (Figure 1). L929 cell viability was reduced as expected to 52% in medium without FBS.

### 3.2. Evaluation of Plasminogen on Cell Migration

**Figure 2:**
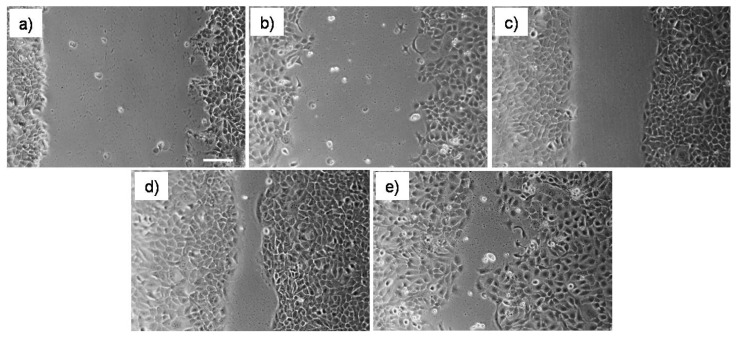
In (**a**) the scratch at time 0. At 20 h, untreated wounds showed about 20% closure without FBS (**b**) and about 53% closure with FBS (**c**). At 20 h, 64 nM Plg treated wounds with (**d**) and without FBS (**e**) are almost healed. Scale bar = 200 mm. Original magnification 4×.

We investigated whether Plg treatment may affect keratinocyte proliferation and migration. In the scratch cell migration assay, Plg promoted wound recovery (Figure 2). The addition of Plg to medium culture was able to prompt the cell migration on scratch test using HaCaT cells. Cells treated with 64 nM Plg in both complete and serum-free medium showed faster scratch closure (about 80 and 74% respectively) than in complete medium after 20 h from scratch (about 53%).

### 3.3. Scaffold Morphological Characterization

**Figure 3:**
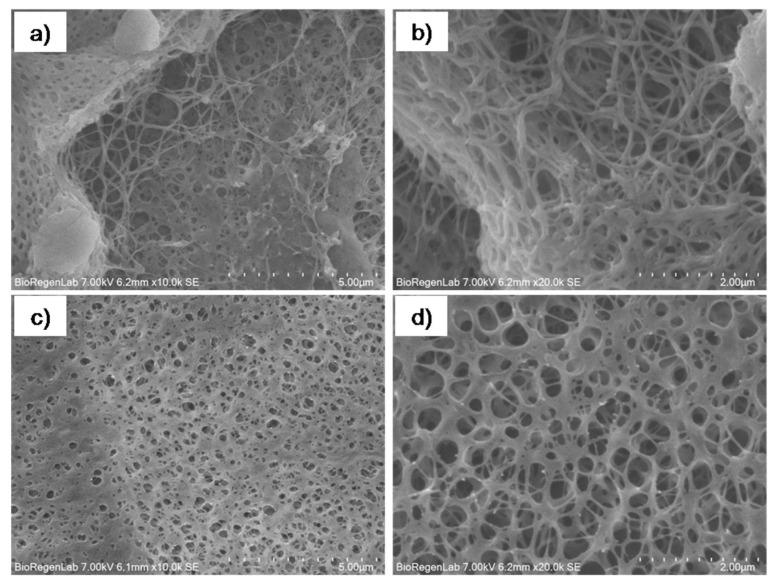
SEM images (**a**,**b**) of the exposed side and (**c**,**d**) of the side in contact with the mandrel 10,000× and 20,000×.

SEM images showed the typical nanofibrillar structure of fibrin on the scaffold air contacting surface (Figure 3a,b). The diameter of the fibrin fibers on the air contacting surface was uniform (about 80 nm), the fiber nodes were few, the fiber filament interweaved and overlapped to form a fiber network, the spatial structure was relatively dense. Instead, a compact microporous structure (pores diameters = 394 ± 84 nm) was observed on the mandrel contacting surface (Figure 3c,d).

### 3.4. Scaffold Mechanical Characterization

**Figure 4:**
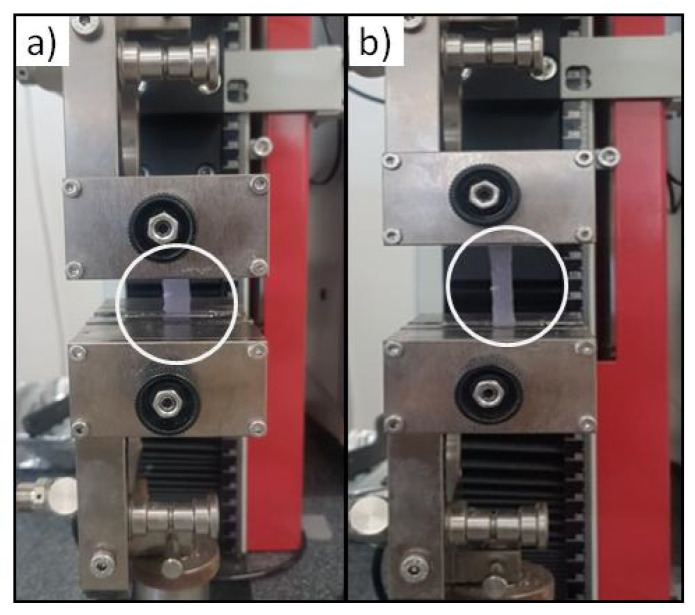
Uniaxial tensile test on Plg-loaded fibrin scaffold (30 × 5 mm) at the beginning (**a**) and during the test (**b**). The white circle highlights the sample.

The fibrin scaffolds Young’s modulus is 59.5 ± 5.6 kPa and it is higher than the fibrin clot modulus, 41.1 ± 11.7 kPa. However, the ultimate stress and strain of fibrin scaffold are higher respect to the fibrin clot (Table 1). There are statistical differences among the fibrin scaffold and clot samples, while no statistical differences were observed between data concerning the Young’s modulus related to fibrin scaffolds loaded and unloaded with Plg. Despite the difference between the stress at break values, the fibrin scaffolds showed the same elastic behavior, which is very dissimilar from fragile fibrin clot behavior. In Figure 4 the elongation of fibrin scaffold during test is shown.

### 3.5. Plasminogen Release Kinetic from Plg-Loaded Fibrin Scaffold

**Figure 5:**
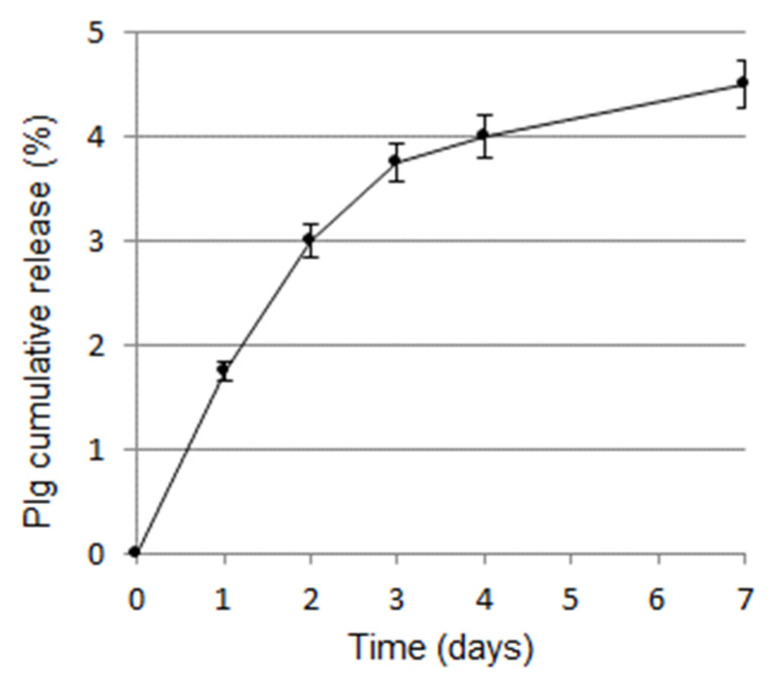
Release kinetic of Plg from Plg-loaded fibrin scaffold.

An initial burst release was observed with Plg-loaded fibrin scaffold, then the release slowed and reached a plateau at 4–7 days (Figure 5). After the incubation time (7 days) scaffold appears to not be degraded without a variation in thickness with respect to time 0.

### 3.6. Wound Area Calculation

The evaluation of Plg-loaded fibrin scaffold efficacy in promoting wound healing was tested in a mouse animal model of full-thickness skin lesion. Significant changes of body weight and blood glucose levels were not observed in any mice during the experimental period. Mean body weight was 44.7 ± 2.1 g at day 0 and 42.9 ± 2.9 g at day 14, while mean glucose value was 434 ± 37 mg/dL at day 0 and 462 ± 41 mg/dL at day 14. At day 7 and 14, only very thin scaffold residues were observed at wound sites in both samples and the secondary dressing was easily removed from the wound.

**Figure 6:**
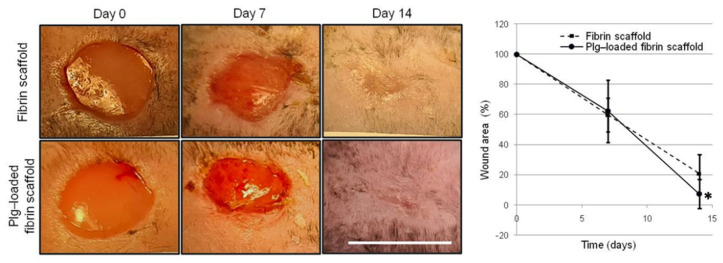
On the left, representative images of lesions treated with fibrin scaffold and Plg-loaded fibrin scaffold at 0, 7 and 14 days (scale bar = 1 cm). On the right, percentage of wound area at 7 and 14 days.

Representative wound images obtained for each treatment group at days 0, 7, and 14 post-wounding are shown in Figure 6. At day 7, no statistical differences were observed in open wound area between fibrin scaffold groups. Plg-loaded fibrin scaffold induced about 93 ± 10% of wound closure in vivo experiment at 14 days, significantly higher with respect to 79 ± 13% observed with Plg-unloaded fibrin scaffold (Figure 6).

### 3.7. Histological Analysis of Wound Healing

**Figure 7:**
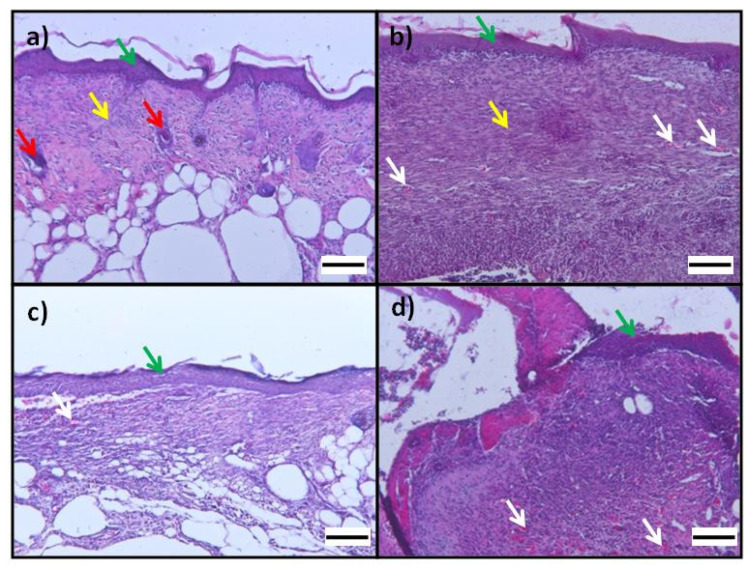
Photomicrographs of wound at day 14: Hematoxylin-eosin histological sections of excisional wound site obtained from a diabetic wound: (**a**,**b**) images from different mice treated with Plg-loaded fibrin scaffolds show epithelialization (green arrow), mild angiogenesis (white arrow), hair follicle (red arrow), mature connective tissue (yellow arrow); (**c**,**d**) images from different mice treated with Plg-unloaded fibrin scaffold show newly formed capillaries (white arrow) in granulation tissue and epithelialization (green arrow); OM 100× (scale bare = 100 µm).

**Figure 8:**
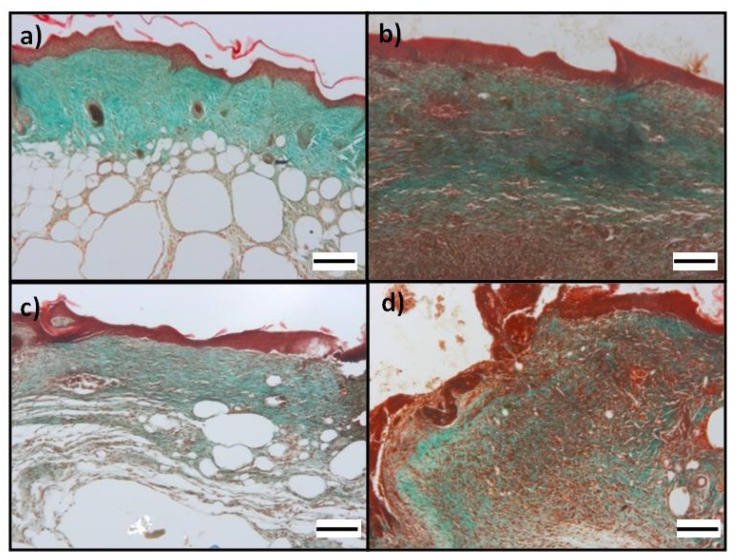
Photomicrographs of wound at day 14: Masson’s trichrome for evaluation of collagen in histological sections of excisional wound site obtained from a diabetic wound: (**a**,**b**) images from different mice treated Plg-loaded fibrin scaffold show a qualitative greater collagen deposition compared to (**c**,**d**) images from different mice treated with Plg-unloaded fibrin scaffold. Collagen fibers stained with light green SF yellowish appear green. All figures show epithelialization and granulation tissue based on the state of wound healing; OM 100× (scale bare = 100 µm).

As evidenced by the histological analysis, all the scaffolds, although to different degrees, induced re-epithelialization, formation/maturity of granulation tissue and collagen deposition (Figure 7 and Figure 8). The fibrin scaffolds made with the spray technology resulted in an excellent wound management system that allows to keep the wound hydrated for a long time.

The application of Plg-loaded fibrin scaffold on full-thickness dorsal skin wounds showed a more complete re-epithelialization (Figure 7) and qualitative higher collagen deposition (Figure 8) in granulation tissue at day 14 with respect to Plg-unloaded fibrin scaffold.

The semiquantitative histopathological scoring showed that Plg-loaded fibrin scaffold induce a significantly higher re-epithelialization (2.5 ± 0.7) and collagen deposition (2.3 ± 0.8) respect to Plg-unloaded fibrin scaffold (1.4 ± 1.3 and 1.3 ±1.2 respectively). Concerning granulation tissue, a higher presence of inflammatory cells was observed in mice treated with Plg-unloaded fibrin scaffold (2.4 ± 1.0) respect to mice treated Plg-loaded fibrin scaffold (1.0 ± 1.3).

## 4. Discussion

Wound healing is a complex process that encompasses three main phases: inflammation, proliferation and tissue remodeling. These events are driven by growth factors and cytokines, which are secreted at the wound site by inflammatory cells and other stromal cells in response to tissue injury. The impaired wound healing is evidenced by increased closure time and decreased in granulation tissue formation and collagen deposition.

Emerging therapies are likely to evolve from a mere restoration of a physiological context toward the stimulation of the regenerative capacity of the damaged tissue. This trend can potentially address the significant unmet needs in chronic wounds treatment, and in particular in the treatment of diabetic ulcers whose incidence is steadily increasing due to the increased global prevalence of diabetes [15].

Recently it has been demonstrated that another important mechanism in wound healing is represented by Plasminogen (Plg) and the Plasminogen activator system (PAs). PAs are a proteolytic system in which the active protease, plasmin, is generated from the conversion of the precursor, Plg, by either of two physiological PAs: tissue-type PAs (tPAs) or urokinase-type PAs (uPAs) [16]. PAs are widely used for the generation of extracellular proteolytic activity. It is well established that the PAs play a pivotal role in fibrinolysis and in many tissue-remodeling processes, including wound healing [16,17].

A relatively recent study has shown that the healing of tympanic membrane perforations is delayed in uPAs-deficient mice and is completely arrested in Plg-deficient mice [18], indicating that the role of the PAs in the wound healing process is more critical than previously appreciated.

A subsequent study of the same group shows that Plg is a key regulatory molecule with a pronounced pro-inflammatory effect that accumulates in the wounded area during the first day of wound healing. It activates signal transduction and potentiates the early inflammatory response. Systemic administration of additional Plg not only accelerates the healing of acute wounds, but it also initiates and improves the healing of chronic diabetic wounds [4]. A study in diabetic patients shows that glycation of Plg leads to decreased plasmin generation and impaired functional protein activity and, as a consequence, impaired fibrinolysis. This occurs through two main mechanisms: decreased conversion of Plg to plasmin and reduced catalytic efficiency of generated plasmin. These findings appear to be related to the preferential glycation sites on Plg [19].

Another study reports that Plg-deficient mice have extensive fibrin and neutrophil depositions in the wounded area long after re-epithelialisation, indicating inefficient debridement and chronic inflammation. Delayed formation of granulation tissue suggests that fibroblast function is impaired in the absence of Plg. Therefore, besides activating inflammation, Plg is also crucial for the subsequent termination of inflammation and activation of the proliferation phase. Supplementation of Plg-deficient mice with human Plg leads to a restored healing process comparable to that in wild-type mice [19].

Taken all together these results suggest that the administration of Plg may be a novel therapeutic strategy to treat many different types of wounds, especially chronic wounds such as those caused by diabetes.

In this work we observed that in cell culture experiments Plg supplied by Kedrion stimulated fibroblasts proliferation and wound recovery in keratinocytes according to literature data [20]. Then we investigated the feasibility to produce by spray technique a Plg delivery system based on fibrin due to our groups previous experiences in biological and synthetic scaffolds.

Fibrin/fibrinogen could be processed to obtain polymeric constructs by different techniques such as 3D bioprinting and electrospinning.

3D bioprinting represents an emerging technology to produce biological scaffolds. However, fibrinogen alone has a Newtonian behavior that limits the possibility to bioprint it as a bioink. Numerous fibrin-based bioinks with tunable bioprinting properties could be achieved combining fibrinogen solution with printable biomaterials [21]. Recently, in-situ crosslinking of fibrinogen-based bioink consisting of a coaxial extrusion system, in which the crosslinker reacts with fibrinogen at the end of the nozzle, allowing for the layer-by-layer deposition of fibrin combined with alginate or hyaluronic acid [22,23].

Fibrin can be also electrospun; however, the methods are not suitable for a drug delivery scaffold preparation since drugs can be released during washing steps or inactivated by glutaraldehyde cross-linking [24,25,26]. Recently our group developed a process that does not required any further treatment, such as washing steps, to produce a bilayered fibrin/polyurethane scaffold loaded with platelet lysate by a combination of electrospinning and spray, phase-inversion method for wound healing. The scaffold is composed of a nanostructured fibrin layer with bioactive function obtained by electrospinning and a polyurethane layer obtained by spray technology to confer mechanical support [13].

The spray-technology is a key element in this study, where a computerized system that controls the implementation of a set-up of two separate ejectors (spray-guns) with convergent jet is employed to assemble a drug delivery system to achieve the therapeutic objective. The spray-technology is characterized by several advantages: (i) the spray process is based on the use of solutions that allow employing the fibrinogen and thrombin solutions as they are commercialized; (ii) the solutions are loaded into two different syringes and the polymerization between fibrinogen and thrombin occurs at the same time of the material deposition on the rotating mandrel; (iii) the fibrin scaffold is characterized by a micropores structure.

The spray process described in this work allows for producing in a single step a bioactive fibrin scaffold with resistant and handling properties, as demonstrated by uniaxial tensile test in comparison with fibrin clot. The chosen flowrates allowed for obtaining a fibrin deposition onto a mandrel without drips and loss of materials.

Hickerson W.L. et al. in 2011 studied the Evicel fibrin sealants in form of clot with fibrinogen 55–85 mg/mL and thrombin 800–1200 IU/mL obtaining a Young Modulus equal to 38 kPa, a value similar to the Young Modulus calculated in this study [27].

The fibrin network observed on to Plg-loaded fibrin scaffold air surface is characteristic of fibrin clot [28,29]. The fiber diameters in the scaffolds surface are similar to fibrin clot fibers described in literature [30], while a peculiar dense structure with micropores due to protofibril aggregation was observed on the mandrel surface. This structure probably confers the higher mechanical properties to scaffold with respect to clot.

The in vitro Plg release kinetics was evaluated by ELISA for up to 7 days. The fibrin scaffold released a burst of Plg on the first 2 days and a gradual constant release was observed up to day 7 (about 5%). Since scaffold appeared to not be degraded at day 7, the results suggest that the Plg release was due to diffusion in aqueous solution.

The components and functions of the murine fibrinolytic system are quite similar to those of humans. It has been demonstrated that murine Plg contains functional binding sites in domains similar to human Plg [31]. The interactions between murine Plg, u-PA are qualitatively similar to those between their human counterparts. However, quantitative differences were observed in u-PA-mediated fibrinolytic activity in the murine systems with respect to humans [32]. In this work Lijnen and colleagues demonstrated that, although quantitative interactions between purified components of the murine fibrinolytic system appear to be comparable to those between the human counterparts, murine plasma clots are >30-fold more resistant to lysis with autologous tissue-type plasminogen activator than human plasma clots.

The in vivo experiments performed in the diabetic mouse wound-healing model showed at scaffold changing (at 7 and 14 days timr-points) that the fibrin layers are almost completely degraded. An excellent outcome on wound closure rate was observed both for Plg-unloaded fibrin scaffold (79% at day 14) and for Plg-loaded fibrin scaffold (93% at day 14). Wound closure was significantly higher in Plg-loaded fibrin scaffold respect to the Plg-unloaded one, however also Plg-unloaded fibrin scaffold obtained by spray technology showed optimal activity in term of wound area reduction. In previous experiments Mepore polyurethane film, used in this study was used as a secondary dressing, was used as control since it does not possess any bioactive properties but it allows to maintain hydration and sterility. In Mepore mice assumed as untreated groups only a 26% of wound closure was observed at days 14 [13]. Moreover, the semiquantitative histopathological analysis showed that Plg-loaded fibrin scaffold induce a significantly higher re-epithelialization and collagen deposition and a lower inflammation respect to Plg-unloaded fibrin scaffold.

Although in the present study in vivo results were obtained in a limited cohort of animals (10 per group) these data demonstrated a clear efficacy of Plg local delivery in wound healing. 

At day 14 post-injury, more complete re-epithelialization and collagen deposition were observed in animals treated with Plg-loaded fibrin scaffold with respect to animals treated with Plg-unloaded fibrin scaffold. These results agree with other studies, in which human Plg intravenously administration (daily for 15 days) in Plg-deficient mice leads to a restored healing process [19].

Although it would be good practice to repeat these experiments in a larger number of animals, the results obtained can pave the way to the use of Plg-loaded fibrin scaffold as a drug delivery system to stimulate the closure of diabetic wounds in humans.

## 5. Patents

IT Patent application pending N. 102021000025664, filed on 7/10/2021.

## Figures and Tables

**Table 1:** Shows the obtained values for the stress and strain of fibrin scaffold, Plg-loaded fibrin scaffold and fibrin clot.

	Fibrin Scaffold	Plg-Loaded Fibrin Scaffold	Fibrin Clot
Young’s Modulus [kPa]	59.5 ± 5.6	61.8 ± 4.8	41.1 ± 11.7
Strain at break [mm/mm]	106.8 ± 10.2	108.4 ± 7.8	62.2 ± 1.3
Stress at break [kPa]	66.6 ± 1.2	72.3 ± 2.6	25.3 ± 3.1

## Data Availability

The data presented in this study are available upon request from the corresponding author.
